# The Pattern of Polymorphism in *Arabidopsis thaliana*

**DOI:** 10.1371/journal.pbio.0030196

**Published:** 2005-05-24

**Authors:** Magnus Nordborg, Tina T Hu, Yoko Ishino, Jinal Jhaveri, Christopher Toomajian, Honggang Zheng, Erica Bakker, Peter Calabrese, Jean Gladstone, Rana Goyal, Mattias Jakobsson, Sung Kim, Yuri Morozov, Badri Padhukasahasram, Vincent Plagnol, Noah A Rosenberg, Chitiksha Shah, Jeffrey D Wall, Jue Wang, Keyan Zhao, Theodore Kalbfleisch, Vincent Schulz, Martin Kreitman, Joy Bergelson

**Affiliations:** **1** Molecular and Computational Biology, University of Southern California, Los Angeles, California, United States of America; **2** Department of Ecology and Evolution, University of Chicago, Chicago, Illinois, United States of America; **3** Department of Cell and Organism Biology, Lund University, Lund, Sweden; **4** Genaissance Pharmaceuticals, New Haven, Connecticut, United States of America; Max Planck Institute of Chemical EcologyGermany

## Abstract

We resequenced 876 short fragments in a sample of 96 individuals of *Arabidopsis thaliana* that included stock center accessions as well as a hierarchical sample from natural populations. Although *A. thaliana* is a selfing weed, the pattern of polymorphism in general agrees with what is expected for a widely distributed, sexually reproducing species. Linkage disequilibrium decays rapidly, within 50 kb. Variation is shared worldwide, although population structure and isolation by distance are evident. The data fail to fit standard neutral models in several ways. There is a genome-wide excess of rare alleles, at least partially due to selection. There is too much variation between genomic regions in the level of polymorphism. The local level of polymorphism is negatively correlated with gene density and positively correlated with segmental duplications. Because the data do not fit theoretical null distributions, attempts to infer natural selection from polymorphism data will require genome-wide surveys of polymorphism in order to identify anomalous regions. Despite this, our data support the utility of *A. thaliana* as a model for evolutionary functional genomics.

## Abstract 2

A systematic global survey of genomic DNA sequence polymorphism in *Arabidopsis thaliana* reveals that standard genetic tests for selection do not apply to this species but supports its status as a model organism.

## Introduction

The field of population genetics has always been heavily influenced by mathematical models. Ever since molecular polymorphism data started to become available, in the form of allozymes [1] or DNA sequences [2], population geneticists have searched for footprints of selection by comparing the patterns of polymorphism in particular genes with the pattern expected under standard neutral models [3,4]. Considerable intellectual effort has gone into estimating model parameters such as the mutation rate *θ,* the recombination rate *ρ,* and the effective population size, *N_e_* [3,5]. However, because of the limited availability of data, it has been difficult to determine whether the underlying models are appropriate. For example, demographic factors such as population structure and growth can cause the genome-wide pattern of variation to deviate from standard neutral models in ways that mimic selection [4,6]. Thus, without knowing whether a standard neutral model describes the pattern of variation in most of the genome, it is difficult to conclude that a particular gene has been under selection.

With the advent of high-throughput genotyping and sequencing, sufficient data for the critical appraisal of standard models are starting to become available, especially in humans [7–9]. Here we report our findings from a systematic survey of genomic DNA sequence polymorphism in *Arabidopsis thaliana,* one of the first in any organism. Our goal was to investigate the pattern of polymorphism in a large sample of individuals, using sufficiently densely spaced loci to obtain insight into the genome-wide haplotype structure of the species.

The scale of our study allows us to describe the pattern of polymorphism with unprecedented accuracy. We begin by describing how variation is distributed, with respect to space (i.e., population structure) as well as with respect to haplotypes (i.e., linkage disequilibrium [LD]). Our set of 96 individuals contained hierarchical population samples in addition to a worldwide collection of stock center accessions (Tables S1 and S2): because of this and the large number of polymorphisms, we are able to answer a number of questions about population structure that previous studies have not been able to address.

In the second part of the paper, we compare the pattern of variation to predictions made by standard population genetics models. The number of loci sequenced is sufficient to investigate the distribution of important summary statistics across the genome rather than simply looking at averages (as is usually done).

## Results/Discussion

### Sequencing

A total of 876 reliable alignments, representing 0.48 Mbp of the genome (or a total over all individuals of ~44 Mbp) was generated. The average sequence length is 583 bp; the average sample size across alignments is 89. Based on the *A. thaliana* genome annotation, the composition of our data, which includes more than 17,000 single nucleotide polymorphisms (SNPs) and insertion/deletion polymorphisms, is 15% intergenic, 55% exon, 22% intron, 4% UTR, and 5% pseudogene (see Materials and Methods). The majority of fragments, 67%, contain both coding and noncoding sequence.

### Population Structure

#### Overall levels of polymorphism

**Figure 1:**
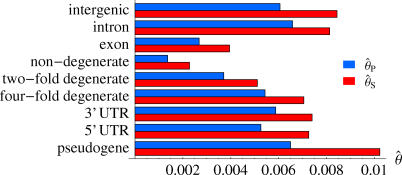
Levels of Polymorphism for Different Classes of Sites Levels of polymorphism were quantified using two different estimators of the neutral mutation rate *θ*: θ^_*S*_ , which uses the number of polymorphic sites, and θ^_P_ , which uses the average number of pairwise differences [3].

Our estimates of the level of polymorphism are broadly comparable to what has previously been found in *A. thaliana* and other species, both in terms of overall levels of polymorphism, and in the degree of constraint on different kinds of sites (Figure 1; cf. [3]). The highly selfing *A. thaliana* does not have unusually low levels of polymorphism: the observed values are somewhat lower than for *Drosophila melanogaster,* and are considerably higher than for humans.

A standard way of summarizing the geographical distribution of this variation is through the statistic *F_ST_,* which, loosely speaking, measures the fraction of the observed genetic variation that is due to population structure [10]. Our sample contains 40 individuals that were hierarchically sampled in pairs from four populations in each of five regions (Table S1). A hierarchical analysis of variation in these individuals reveals that 33% of the global variation is segregating among individuals within populations, 35% is segregating among local populations within regions, and 26% is segregating among regions. Only 6% of the variation in the global sample is not captured by these 40 individuals. Even though only two individuals were sampled per population, and even though our estimates of within-population variance are upwardly biased (individuals were prescreened to avoid sequencing identical individuals; see Materials and Methods), our data clearly show that individual populations harbor much of the variation present species-wide. At the same time, there is strong population structure.

#### Global geographic structure

Studies of variation in *A. thaliana* have typically not found any correlation between the genotype and geographic origin of accessions. This has been attributed to a recent expansion of the species, perhaps in combination with human disturbance. However, early studies had little power to detect population structure, and a more recent, larger survey revealed weak isolation by distance [11]. Our study has several orders of magnitude more markers than any previous study known to us, and we find clear global population structure.

We used a model-based clustering algorithm, implemented in the computer program Structure, to cluster our accessions on the basis of genotype [12]. Loosely speaking, the algorithm attempts to identify a predetermined number of clusters, *K,* that have distinctive allele frequencies, and assigns portions of individual genomes to these clusters. A genome can have membership in several clusters, and the algorithm reports the probability distribution of the assignment of each section of the genome.

**Figure 2:**
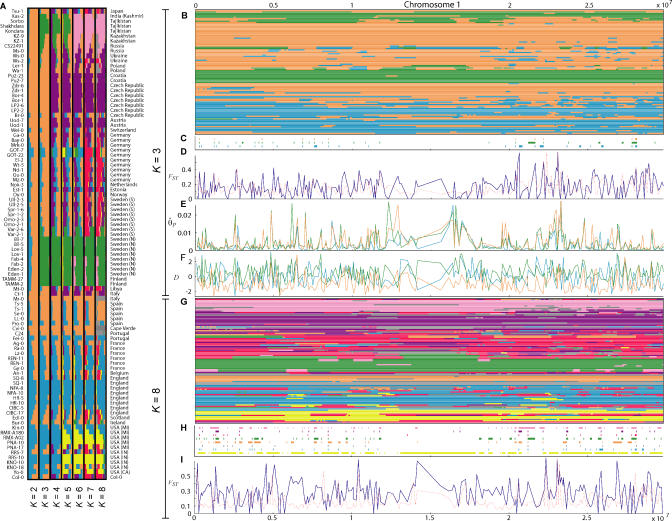
Population Structure and Genomic Distributions of Various Statistics (A) Results from Structure under different assumptions about the number of clusters (*K* = 2,…, 8). Each individual is represented by a line, which is partitioned into *K* colored segments according to the individual's estimated membership fractions in each of the *K* clusters. The assignment of each individual is the average across the genome. (B) Results from Structure across Chromosome 1 for *K* = 3. Each chromosomal segment is colored according to the cluster in which it had the highest probability of membership. (C) A plot showing those fragments that appear to be monophyletic with respect to each of the three clusters identified by Structure. (D) *F_ST_* with respect to the same three clusters (blue solid line) and the lower 95th percentile of *F_ST_* obtained through 1,000 random permutations of the accessions (red dotted line). (E) θ^*_P_* within each of the three clusters. (F) Tajima's *D* statistic within each of the three clusters. (G) Results from Structure across Chromosome 1 for *K* = 8. (H) A plot showing those fragments that appear to be monophyletic with respect to each of these eight clusters. (I) *F_ST_* with respect to these eight clusters.

We analyzed the data by successively increasing *K* from two to eight (Figure 2). For *K* = 2, we see an East–West gradient, potentially attributable to post-glaciation colonization routes [11]. When we increase*K* to three, all accessions from northern Sweden and Finland are assigned to a single cluster together with (to varying degrees) accessions from Eastern Europe, Russia, and Central Asia. While a relationship between northern Sweden and Tajikistan may seem far-fetched, several species are known to have colonized the Scandinavian Peninsula from both north (from Russia via Finland) and south (from Europe via Denmark) after the last glaciation [13].

As we increase *K* from three to eight, each new cluster splits a previously existing one along plausible geographical boundaries (and identifies some accessions as mixed). Thus *K* = 4 separates central European (Czech, Austrian, and Croatian) accessions from the main European cluster, *K* = 5 separates a subset of the United States accessions from the rest of Western Europe, *K* = 6 separates the Central Asian and Russian accessions from northern Sweden and Finland, *K* = 7 separates many German and southern Swedish accessions from the rest of Europe, and *K* = 8 identifies a Catalan cluster. Clusters identified for *K* > 8 did not contain the majority of any single individual genome.

**Figure 3:**
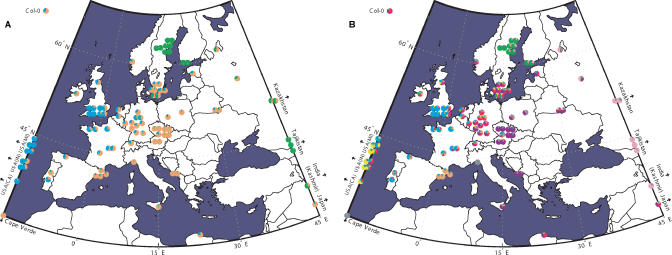
Population Structure in *A. thaliana* Each pie chart represents an accession, and is placed on the map according to origin (some of the population samples were too densely sampled and have been shifted for clarity). Accessions sampled outside Europe have been placed at the correct latitude. The exact origin of the standard lab accession Col-0 is not known. The colors and proportions within each pie chart correspond to the output of Structure in Figure 2. (A) *K* = 3; (B) *K* = 8.

When these results are superimposed on a map (Figure 3), the pattern of isolation by distance becomes obvious. Individuals are, by and large, more similar to individuals that grow nearby than to individuals from far away. Although *A. thaliana* commonly occurs as a weed and human commensal, this has not been sufficient to erase population structure.

**Figure 4:**
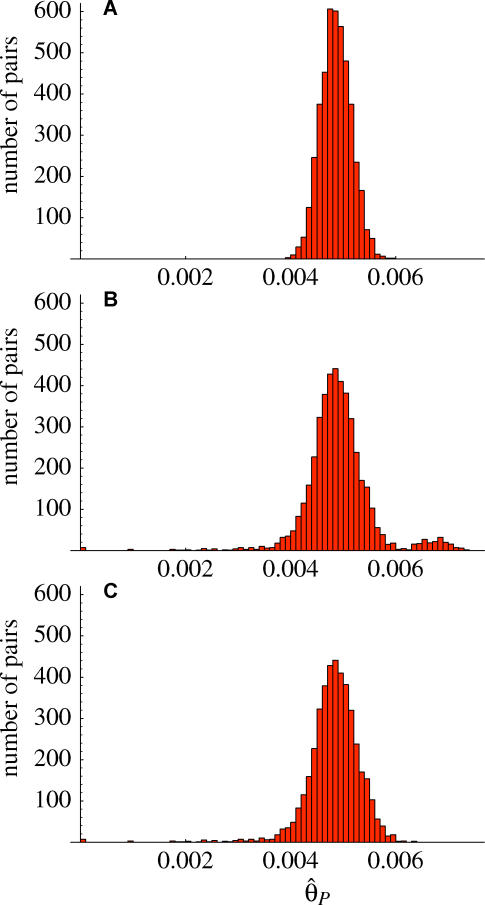
The Distribution of Pairwise Differences (SNPs Only) between All Pairs of Accessions (A) An example of the distribution we would expect to see in the absence of population structure, obtained by randomizing genotypes with respect to individuals for each sequenced fragment. (B) The observed distribution. (C) The observed distribution with accessions Cvi-0 and Mr-0 removed.

Population structure is also evident in the distribution of pairwise differences between individuals. In the absence of population structure, all individuals should be equally closely related on average. As shown in Figure 4, this is clearly not the case. Not only is there generally a wider range of variation than would be expected in the absence of population structure, but there are also clear outliers. Some individuals are extremely closely related: these are typically from the same local population (and will be further discussed below). Perhaps more surprising is that two stock center accessions, Cvi-0 (Cape Verde Islands) (cf. [14]) and Mr-0 (Italy), are very different from all others (and each other).

The distribution of pairwise differences can be conveniently summarized using hierarchical clustering. The population structure revealed by the resulting tree (Figure S1) generally agrees with the output of Structure.

#### Variation within and between populations

Because it is highly selfing, *A. thaliana* has often been considered a collection of asexual lineages, or “ecotypes.” This view is completely false. Indeed, even the first sequencing survey of variation showed clear evidence of recombination between accessions [15], and a subsequent study has shown that recombination has generally been sufficient to erode genome-wide LD on a very fine scale [16]. Consequently, there is no “phylogeny” of ecotypes.

However, this still leaves open the possibility that local population structure is tree-like, with individuals within the same population being much more closely related to each other than to individuals from other populations. Indeed, since *A. thaliana* is a selfer, it is perfectly possible for a local population to consist of a single inbred sibship.

We find that this is typically not the case; most sampled populations were polymorphic (even though this part of our study had relatively little power; see Materials and Methods), and when we consider the genome-wide data, the pattern that emerges is far from tree-like. As shown in Figure 2C and 2H, only a small fraction of all sequenced fragments have patterns of polymorphism compatible with monophyly in the sense that, with respect to that fragment, the members of a particular cluster are all more closely related to each other than to members of other clusters (the yellow “US” cluster is an exception to which we return below). Instead, just as is the case for human populations [17], most polymorphisms are shared between clusters, and levels of polymorphism are in general comparable within all clusters (see Figure 2E; for clarity, only values for *K* = 3 are shown).

**Figure 5:**
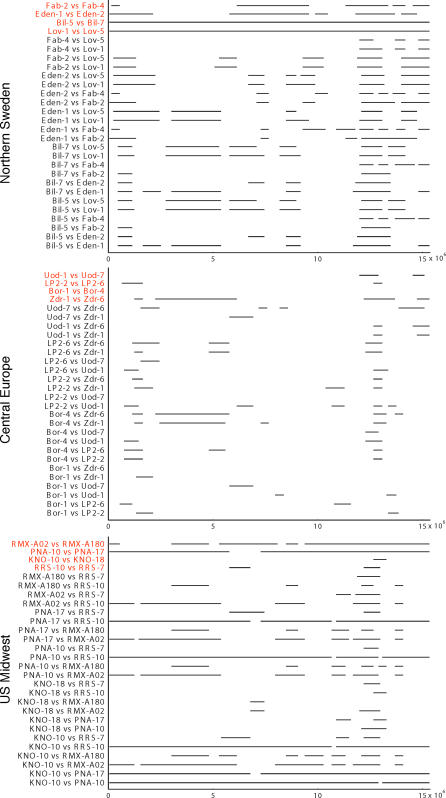
Haplotype Sharing on Chromosome 4 among Pairs of Individuals in the Population Samples from Northern Sweden, Central Europe, and the US The lines indicate regions where the particular pair of accessions share at least five identical adjacent fragments. Within-population comparisons are highlighted in red. The patterns in southern Sweden and in the UK are similar to that in central Europe.

The same is true with respect to the local populations, although there is great variation between regions. An interesting way to describe the relationship between individuals is to try to identify chromosomal regions shared identical by descent by looking for long identical haplotypes [18–22]. The resulting patterns clearly reveal the difference in structure between geographic regions (Figure 5). In northern Sweden, pairs of accessions sampled from the same population are invariably more closely related to each other than to accessions from other populations; however, even here populations are far from monophyletic in the sense used above (i.e., for a particular locus, the closest relative may well come from a different population). In most regions (exemplified in Figure 5 by central Europe), haplotype sharing is moderate, and is not much greater within than between populations. The US sample is again different: here, pairs of accessions often appear to share entire chromosomes, and are equally likely to do so between populations.

Regional variation in the level of population structure is also evident in the distribution of pairwise differences (see Figures 4 and S1). Individuals that are extremely closely related (less than ten differences) are almost always pairs from the same local population (two pairs from northern Sweden, one pair from Finland, and one from Germany). The one exception is a trio of nearly identical individuals from different US populations.

Figure 2D and 2I illustrate the variation in *F_ST_* across the genome for *K* = 3 and *K* = 8. This pattern is of interest in that regions with extremely high or low values may be seen as candidates for harboring selectively important loci [23,24].

#### Structure summarized

**Figure 6:**
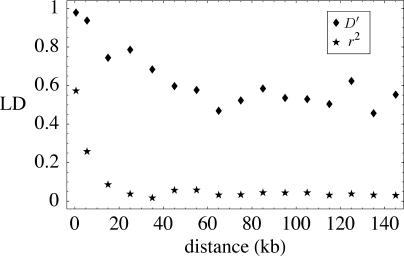
The Decay of LD as a Function of Distance between the Polymorphisms

The picture that emerges is that of a single, large, globally distributed population with historical gene flow sufficient to ensure that variation is shared worldwide, yet limited enough to cause considerable population structure. Genetic exchange is not only geographic; there has been enough outcrossing to ensure that LD decays within 25–50 kb on average (Figure 6), which is comparable to what has been observed in humans. All of this may seem surprising given the highly selfing nature of *A. thaliana,* but it is in fact completely compatible with theoretical predictions [16,25].

The only exception to this pattern comes from the US. Our sample from the US Midwest is clearly a heterogeneous collection, characterized by genome-wide LD and haplotype sharing. Especially notable is extensive haplotype sharing with accessions from other regions, in particular with the United Kingdom. All this strongly suggests that *A. thaliana* is a recent human introduction to the New World, and that the introduction severely reduced haplotype variation through bottleneck effects, causing genome-wide LD [16]. Since we have population samples from only the Midwest, we cannot rule out the possibility that the pattern is different in other parts of the US; however, we note that our one non-Midwestern US accession, Yo-0 (from Yosemite, California), is almost identical to some of our Midwestern accessions, and that a recent survey of variation in 53 US populations found no evidence of differentiation across the continent [26].

It should be emphasized that we view both Structure and hierarchical clustering as tools for exploring the data. The results should not be taken literally. For example, we do not believe that there are *K* = 8 random-mating populations in *A. thaliana,* as might be suggested by Figures 2 and 3, nor do we believe that populations are related in a tree-like manner, as might be suggested by Figure S1.

### Global Patterns

#### Allele frequency distribution

**Figure 7:**
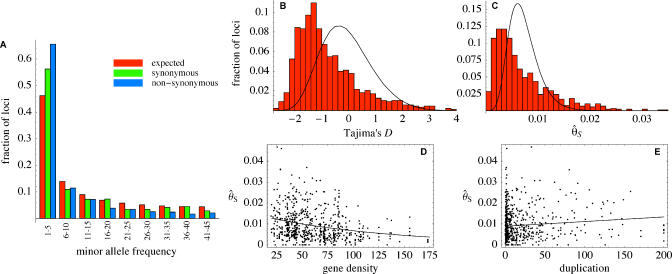
Characteristics of the Pattern of Polymorphism (A) The allele frequency distribution for synonymous and nonsynonymous SNPs using a sample size of 90 individuals (loci with less than 90 individuals were not used; loci with greater than 90 individuals were randomly culled). For a sample of size *n,* the expected frequency of SNP loci with a minor allele frequency of *i* under a standard constant-size population genetics model is 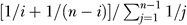 . The excess of rare alleles is largely limited to frequencies one and two. (B) The distribution of Tajima's *D* statistic [27] across the sequenced fragments, along with its expected distribution in a constant population (estimated by simulating 1,000 datasets matching the real one in terms of exon/nonexon composition and sample size). (C) The distribution of the level of polymorphism (θ^*_S_* ) across the sequenced fragments along with its expected distribution (estimated the same way). (D) The level of polymorphism in nonexon sequences as a function of the local gene density (measured in open reading frames per centimorgan). (E) The level of polymorphism in nonexon sequences as a function of the degree of duplication in each fragment (measured as the negative log_10_ of the BLAST significance for the second-best hit in the genome). The patterns in (D) and (E) are also seen in exons.

Note that *θ^_S_* is consistently higher than *θ^_P_* (see Figure 1). This is typically caused by an unusually high ratio of rare to common alleles, compared to what is expected under standard population genetics models [27]. A closer look at the distribution of allele frequencies reveals that this is indeed the case (Figure 7A). The observed distribution is skewed toward rare alleles compared to standard neutral expectations. A possible explanation for this is recent population growth [28]; however, the skew is much greater for nonsynonymous than for synonymous polymorphisms, suggesting that selective factors must also be involved.

The effect of this genome-wide deviation from standard neutral models on “tests of selection” can be dramatic. These tests typically assume that the standard neutral model describes most of the genome, and interpret deviations at particular loci as signs of selection [3]. Our results show clearly that this procedure is not appropriate for *A. thaliana* (see also [29]).

It is, of course, not a new finding that demographic history can invalidate tests of selection, but our results provide a striking illustration of the potential seriousness of the problem. For example, the mean value of one popular statistic, Tajima's *D* [27], is −0.8 rather than the (approximately) zero expected under simple neutral models, and the variance is also larger than predicted (Figure 7B). Positive values of Tajima's *D* are typically attributed to balancing selection: 2% of our fragments are significantly positive at the 1% level. Negative values are typically attributed to directional selection: 15% of our fragments are significantly negative at the 1% level. Although some of these deviations may, of course, actually be due to selection, our data suggest that tests based on standard cutoff values are anticonservative in both tails of the distribution. Consistent with this interpretation, a much higher fraction of studied genes have been reported to be under selection in *A. thaliana* than in other species [30].

#### Variation in the level of polymorphism

While it is straightforward to fit a neutral model with growth to the observed distribution of Tajima's *D* (Figure 7B), the value of this exercise is doubtful. First, it is clear from Figure 7A that selection must be part of the reason for the skewed allele frequency distribution. Second, a model with growth would, in fact, fit other aspects of the data less well. In particular, population growth tends to reduce the variability in coalescence times across the genome compared to models with constant population size, resulting in less variation in the level of polymorphism between loci. We see the opposite: the variance between loci is considerably greater than expected under a standard neutral model with constant population size (Figure 7C). In addition, the distribution is heavily skewed and displays a long tail of extremely high values.

There are several reasons to expect a poor fit to a simple neutral model. One is variation across the genome in the “neutral” mutation rate, *θ*, either due to variation in the level of selective constraint or due to variation in the actual, underlying mutation rate. Since the excess variability is observed equally for coding and noncoding DNA, we would have to invoke the latter. The extent to which variation in the mutation rate contributes to the pattern in Figure 7C can be estimated as soon as divergence data from a closely related outgroup species become available.

Another factor likely to contribute to the variation in the level of polymorphism is population structure such as that described in the first part of the paper. It is well known that population structure can inflate the variance of coalescence times as well as induce a tail of very large values that would result in patterns of variability such as the one observed [6]. However, strong population structure is generally expected to push the distribution of Tajima's *D* toward positive values, which is the opposite of what we observe. It is possible that some model that involves growth (perhaps in conjunction with bottlenecks), structure, and finite sampling [7] could explain the pattern observed in *A. thaliana,* but we have not been able to find such a model (see also [29]).

#### Genomic patterns of polymorphism

It turns out that the pattern of polymorphism is affected by not only demographic forces, but also factors intrinsic to the genome. Figure 7D shows that polymorphism in noncoding regions is negatively correlated with local gene density. This mirrors the positive correlation between polymorphism and local recombination rates that was first noted in *Drosophila* [31] and has since been observed in a wide range of organisms [32]. A possible explanation is that recombination itself is mutagenic: this appears to explain the correlation observed in humans, but is not sufficient to explain the phenomenon in general [32,33]. Instead, it has been proposed that variation is reduced in low-recombination regions because of a “hitchhiking effect” due to selection on linked sites [34,35], in the form of either positive selection (“selective sweeps”) [36] or purifying selection (“background selection”) [37]. Such hitchhiking effects would be stronger in low-recombination regions because sites in these regions are affected by selection on larger pieces of the chromosome (i.e., more genes). Recombination is thus used as a proxy for gene density: the real prediction of these models is that polymorphism should decrease with gene density. This is precisely what we observe. The level of polymorphism is insignificantly positively correlated with recombination (suggesting that although recombination may well be mutagenic, this does not explain the phenomenon), but is strongly negatively correlated with gene density.

Two factors suggest that background selection rather than selective sweeps is responsible for the correlation. First, unlike background selection, selective sweeps are expected to skew Tajima's *D* toward negative values. However, we find no correlation between Tajima's *D* and gene density. Second, it is clear from Figure 7A that a significant load of deleterious mutations (as is required by background selection) exists in *A. thaliana*.

Figure 7E reveals that polymorphism is also positively correlated with segmental duplication, similar to what has been observed in humans [38–40]. In humans, the phenomenon appears to be largely due to misclassification of paralogous sequence variants as SNPs. Distinguishing between paralogous sequence variants and true SNPs is difficult for human data, where putative SNPs are typically detected in small samples of highly heterozygous individuals. In contrast, our data consist of high-quality sequences from a large sample of almost completely homozygous individuals, and we are therefore confident that nearly all of our polymorphisms are genuine (see Materials and Methods); fragments that have a close match elsewhere in the genome thus appear to be more variable than fragments that do not. We hypothesize that this is caused by a low level of intergenic gene conversion that serves to “shuffle” variation between loci. Such gene conversion has long been known to occur in large multigene families (“concerted evolution”; [41]): our results suggest that it may be a general phenomenon.

#### Recombination and gene conversion

We noted above (see Figure 6) that LD decays within 25–50 kb, somewhat faster than has previously been suggested [16]. At least 25% of our sequenced fragments show evidence of recombination (using the four-gamete test; [42]). Estimates based on coalescent models suggest an effective population recombination rate (e.g., [6]) of approximately *ρ* = 2 × 10^−4^ per basepair (V. P., B. P., P. Marjoram, J. W., and M. N., unpublished data). Given our estimates of the mutation rate *θ* (see Figure 1), this implies a ratio *θ/ρ* of about 20.

The short-range pattern of LD in several species is incompatible with the long-range pattern; there is too little of the former relative to the latter for a simple recombination model that includes only crossing over to explain the data [43–48]. Possible explanations include gene conversion and multiple mutations (i.e., each SNP not being due to a unique mutation event), both of which will erode short-range LD [46]. There is clear evidence for both phenomena in our data. We observed a total of 315 tri-allelic SNPs. Since less than 50% of all multiple-hit mutations will result in more than two distinct alleles, this suggests that a total of more than 600 of our SNPs are, in fact, the product of multiple mutations. We also observed three fragments that show clear evidence for gene conversion in that a single gene conversion event (i.e., a double cross-over within 500 bp, as would result from the resolution of a single Holiday junction) suffices to explain a complicated pattern of polymorphisms based on multiple SNPs in the fragment. Coalescent-based analyses based on the fine-scale pattern of polymorphism suggest that gene conversion is about five times more common than crossing over, in agreement with previous population genetic analyses [48], as well as with direct estimates based on tetrad analysis [49].

### Concluding Remarks

We have shown that the pattern of polymorphism in *A. thaliana,* a selfing human commensal, generally agrees with what would be expected for a widely distributed sexually reproducing species. Although there is significant population structure, polymorphism is shared worldwide. As predicted by population genetics theory [25], the only clear indications of selfing in the pattern of polymorphism are that individuals are typically homozygous, and that LD is unusually extensive.

The scale of our study allows us to consider the genomic distribution of statistics commonly used to summarize polymorphism data. We find that these distributions generally deviate significantly from what is assumed by standard population genetics models. This highlights the danger of using highly parameterized models based on untested assumptions for inference in population genetics. Commonly used “tests of selection” are simply not valid in *A. thaliana* (cf. [29,30]). Large-scale analyses in other organisms have similarly found genome-wide deviations from standard models (e.g., [43,50,51]). As data continue to accumulate, the focus of population geneticists will surely have to shift from rejecting null models that do not fit particular loci to finding models that actually do explain the bulk of the data.

Genomic polymorphism data are required to develop more robust inference methods, and will enable us to study phenomena that are intrinsically genomic (e.g., the correlations in Figure 7D and 7E). More importantly, however, these data will help identify the functional polymorphisms that underlie phenotypic variation. The pattern of polymorphism in *A. thaliana,* characterized by humanlike levels of LD but much higher SNP density, coupled with the availability of naturally occurring inbred lines, makes the species ideal for LD mapping. Although the strong population structure is likely to cause a high rate of spurious genotype–phenotype associations, these problems can easily be overcome through direct experimental verification using crosses or transgenics. This significantly strengthens the position of *A. thaliana* as a model for evolutionary functional genomics.

## Materials and Methods

### Sampling

The sample of 96 individuals included pairs of individuals from 25 local “populations” (typically sampled within a few hundred meters of each other, often much closer) as well as a worldwide survey of commonly used stock center accessions (Tables S1 and S2). Where possible, four populations were sampled from each of several regions.

The sample was generated by screening a larger set of accessions with a small number of markers to avoid inbred siblings or extensively heterozygous individuals (E. B., E. Stahl, C. T., M. N., M. K., and J. B., unpublished data). Accessions were genotyped using 11 unlinked markers (five microsatellites, two indel R-genes, and four housekeeping genes with previously identified polymorphisms). To ensure that individuals sampled from local populations were not part of inbred sibships, four (three in one case) individuals from each of 37 populations were tested. Polymorphism was found in 25 of these populations, and a pair of nonidentical individuals was selected at random from each (Table S1). Some accessions not from the same population were also found to be identical with respect to these markers (Col-0 and Lp2-2; Ts-1 and Shahdara), but these were included nonetheless. Five accessions were found to be heterozygous and were eliminated. Four of these were from the population samples, and one, Ms-0, was from the stock center. Further testing of two additional Ms-0 lines revealed one more heterozygote and one homozygote, which was included. In spite of these precautions, one sequenced stock center accession, Van-0, turned out to be extensively heterozygous and was eliminated from the analyses in this paper (bringing the sample size to 95).

### Data generation

We used direct, PCR-based sequencing of genomic DNA, with primers designed from the *A. thaliana* reference sequence to cover the genome relatively uniformly. To achieve uniform density of our fragments, the reference genome (releases January 7, 2002, and April 17, 2003) was first divided into equally spaced regions. The last 10 kb of each region then served as an input record to Primer3 (v. 0.6). The designed primer pairs returned from Primer3 for each region were then screened for uniqueness and quality. To screen for uniqueness, all primer pairs were BLASTed (BLAST v. 2.2.3) against both the reference genome as well as BAC datasets downloaded from the Arabidopsis Information Resource (http://www.arabidopsis.org/). Any primer pair that produced a hit in the same region (≤2,300 bps) was removed. Self-amplifying primers were also removed based on this same criterion. Additionally, primers with more than five BLAST hits against the reference were also discarded. To improve the quality of each fragment, any primer pair that amplified a target sequence that contained a homonucleotide run of nine bases or more was removed.

All sequencing was done using ABI 3700 automated sequencers (Applied Biosystems, Foster City, California, United States). All fragments were sequenced in both directions.

Chromatograms were initially base-called with Phred (v. 0.020425.c) and trimmed based on quality value. The start and end of each read was trimmed until the average quality value was 25 in a window of ten bases, and internal bases were converted to missing data when their quality value was below ten. Accessions missing one read of data were trimmed more severely (different setting were used). A combination of Phrap (v. 0.020425.c) and ClustalW (v. 1.82) was used for producing alignments using a modified weight matrix that allowed us to incorporate quality values into the ClustalW algorithm. Alignments were then visually inspected and adjusted as necessary using Consed (v. 13.0). Polyphred (v. 4.20) was used to flag potential heterozygotes, which were confirmed by visual inspection of chromatograms.

Additional trimming was performed as necessary for accessions with multiple false polymorphisms and low-quality sequence after a visual inspection of chromatograms. Whenever two reads from the same accession disagreed, the final call was made by visually inspecting chromatograms unless the difference in quality value made the final call obvious.

Potential polymorphisms in each alignment were then verified by a second person. All alleles found only once or twice in the sample were verified by visually inspecting the chromatograms. When this inspection did not reveal a chromatogram peak clearly different from the other accessions, the base was changed to missing data. This would, if anything, produce a slight underestimate in alleles of frequency one and two. Higher-frequency polymorphisms with generally low-quality values (20 or lower) were also verified by checking the chromatograms.

A total of 876 high-quality fragment alignments were obtained from 979 PCR primers and used for the analyses in this paper. Of the remaining PCR primers, some failed at the stage of PCR amplification and sequencing, while some produced sequencing output that could not be base-called with certainty when the sequence quality was particularly low or when there was evidence that the primer pairs amplified two or more different products in some of the accessions.

To calculate genetic distances, we used a set of markers that have been genetically mapped to the Lister and Dean recombinant inbred lines and that also can be mapped to the AGI reference genome. Some markers were removed so that both physical position and genetic position were monotonically increasing functions.

All data are publicly available through our Web site (http://walnut.usc.edu/2010, and also as Dataset S1.

### Population structure

To infer population structure and assign accessions to populations, we used a model-based clustering algorithm implemented in Structure v. 2.0 [12]. Since *A. thaliana* is largely homozygous, we used a haploid setting. We used the “linkage model” with “correlated allele frequencies” in Structure, where genetic distances (calculated by fitting a third-order polynomial to the Lister and Dean recombinant inbred mapping data) were used to indicate locus proximity. The algorithm was run with a burn-in length of 50,000 MCMC iterations and then 20,000 iterations for estimating the parameters. This was repeated ten times for each *K* (ranging from one to 17). In these analyses, each fragment-haplotype was treated as a marker at a multiallelic locus, so that two accessions had a different type if they differed at any site in the fragment.

The likelihood of the data increases with *K* from *K* = 1 until *K* = 7 (using the Wilcoxon two-sample test to compare the ten runs for each *K*; two-sided *p =* 0.001 for *K* = 7 versus *K* = 6). The likelihoods of *K* = 7 and *K* = 8 were similar (two-sided *p =* 0.97). For *K* > 7, the likelihoods of different runs were more variable than for *K* ≤ 7, with the added variability caused only by runs with lower likelihoods. Moreover, the additional clusters for *K* > 8 do not have a majority of the genome for any of the accessions. These observations taken together indicate that it is less meaningful to choose *K* > 8.

In displaying the output from Structure, we computed an average of the ten runs for each *K*. Because there are *K*! distinct permutations of the clusters that all correspond to equivalent assignments of membership coefficients to accessions, and because independent runs may produce different permutations, to compute an average we first permuted the clusters to align the solutions. For *R* runs, there are (*K*!)*^R^* ^− 1^ ways of aligning clusters across runs. To determine which of the clusters of each of the other runs corresponds to a specific cluster in a given run, the symmetric similarity coefficient (SSC) was used with the matrices of membership coefficients (based on the genome-wide average). For a given *K,* the SSC was calculated for all combinations of pairs of runs:



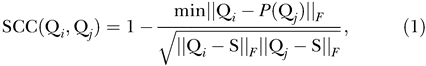



where Q*_i_* and Q*_j_* are the membership matrices of runs *i* and *j* (*i* ≠ *j*), *P* is a permutation; the minimum is taken over all permutations, S is a probability matrix of *K* columns where all elements equal 1/*K,* and A *_F_* is the Frobenius matrix norm [52]. This is a slight adaptation of the asymmetric similarity coefficient used in previous work [17].

For *K* = 2, the runs were permuted to the arrangement that maximizes the sum of SSC across pairs of runs, and an average of the membership matrices across runs was then taken. For *K* > 2, it was not feasible to test all possible arrangements; therefore, the following greedy algorithm was used. (1) Fix a permutation, *P*_1_, of one (randomly chosen) run, 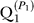 . (2) Randomly choose a second run, Q_2_, and fix the permutation, *P*_2_, that maximizes 
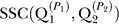
 . (3) Continue sequentially with each remaining run, Q*_x_*, where *x* = 3,…, *R*, and fix the permutation, *P_x_*, that maximizes 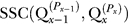 for the current run, Q*_x_*. Because the choice of starting run can affect the result, we tested all ten possibilities for the starting run. For *K* = 2 to *K =* 8, there were thus 70 possible ways of starting the algorithm, and in only two of 70 possible cases was a different result obtained. These two solutions differed from the common solution by switching one pair of clusters in one run (2.5% of the clusters differed from the common solution), and switching one pair of clusters in two different runs (5%).

We tested for monophyly as follows. For every variable site in a fragment, each cluster was checked for the presence of both alleles as well as for the presence of both alleles outside the cluster. If a variable site in a fragment had both alleles within the cluster as well as outside the cluster, then the whole fragment was deemed nonmonophyletic for that specific cluster. Clusters that failed to show nonmonophyly for a fragment were considered monophyletic for that fragment. Fragments with less than five variable sites and clusters with less than five accessions were always considered to be nonmonophyletic.

*F_ST_* for the inferred clusters was computed as:



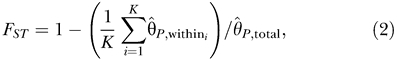



where θ^_P, total_ is the average number of pairwise differences per site for all pairs of accessions, and *θ^_P,_*_within_ is the average number of pairwise differences per site for all pairs within cluster *i*.

Of the total of 95 accessions, 40 were hierarchically sampled in pairs from four populations in each of five regions (Table S1). The total amount of variation among these 40 accessions, *θ^_P,_*_among40_ , was computed by taking the total average pairwise difference for all pairs of the 40 accessions, whereas the amount of variation within populations, *θ^_P,_*_withinpop_ , was calculated by taking the mean of the total average pairwise difference for the pairs of accessions in the 20 populations. The level of variation among geographical regions, *θ^_P,_*_amongreg_ , was computed as the difference between *θ^_P,_*_among40_ and the mean of the total average pairwise differences for all pairs of accessions within regions. The level of variation among populations, *θ^_P,_*_amongpop_ , was calculated from the following expression:



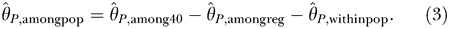



### Genomic patterns of polymorphism

Correlations were identified between levels of polymorphism and local gene density or degree of duplication (Figure 7). The local gene density was measured as open reading frames per centimorgan in windows of size greater than or equal to 1 Mb (using genetically mapped markers from the Lister and Dean recombinant inbred data as endpoints). The number of open reading frames (excluding pseudogenes and RNA genes) from the annotated reference sequence that fell between these window endpoints was counted, and length in centimorgans of each window was estimated from the genetic distance of the markers used as window endpoints.

Correlations were quantified using Spearman's rank correlation, and the significance of the observed values was evaluated using 50,000 permutations that maintained the chromosomal order of all observations but that shuffled the relative positions of the two variables. (For each variable, the lists representing the consecutive values within each chromosome were concatenated in random order and direction to form a circle. The two circles were then randomly aligned with each other.) This is necessary to avoid inflated significance values due to autocorrelations along the chromosomes (of both variables). Using this procedure, the rank correlation between *θ^_S_* in nonexon sequences and gene density is −0.27 (*p* = 0.0014), and the rank correlation between *θ^_S_* in nonexon sequences and the negative log of the second-best BLAST e-value is 0.13 (*p* = 0.0018).

To investigate the effect of population structure, all analyses (except those of population structure) were repeated with the outliers in Figure 4 removed (Cvi-0, Mr-0, and all but one randomly chosen member of each closely related group). All conclusions remain qualitatively the same.

## Supporting Information

Dataset S1 All Data Used in the Paper (912 KB ZIP).

Figure S1 Hierarchical Clustering of Individuals Based on Pairwise Differences (12 MB EPS).

Table S1 The Population Samples Used in the Project (8 KB PDF).

Table S2 The Individual Accessions Used in the Project (8 KB PDF).

## Abbreviations

LD: linkage disequilibrium
SNP: single nucleotide polymorphism
SSC: symmetric similarity coefficient
