# Supplementary material for: The Pattern of Polymorphism in Arabidopsis thaliana
Source: PLoS Biol. 2005 May 24;3(7):e196. doi: 10.1371/journal.pbio.0030196 (PMC1135296; doi:10.1371/journal.pbio.0030196)
Supplement: Supplementary file 1 [file pbio.0030196.st001.pdf]

Table S1. The accessions used in the project: population samples

accession	region	population	latitude	longitude	origin	stock center
Eden-1	N. Sweden	Eden	62.53	18.11	M. Nordborg	CS22572
Eden-2			62.53	18.11	M. Nordborg	CS22573
Lov-1		Lövvik	62.48	18.05	M. Nordborg	CS22574
Lov-5			62.48	18.05	M. Nordborg	CS22575
Fab-2		Fäberget	63.01	18.19	M. Nordborg	CS22576
Fab-4			63.01	18.19	M. Nordborg	CS22577
Bil-5		Billaberget	63.19	18.29	M. Nordborg	CS22578
Bil-7			63.19	18.29	M. Nordborg	CS22579
Var2-1	S. Sweden	Vårhallarna	55.33	14.2	M. Nordborg	CS22580
Var2-6			55.33	14.2	M. Nordborg	CS22581
Spr1-2		Sprattleboda	56.32	14.29	M. Nordborg	CS22582
Spr1-6			56.32	14.29	M. Nordborg	CS22583
Omo2-1		Östra Möcklö-2	56.12	15.18	M. Nordborg	CS22584
Omo2-3			56.12	15.18	M. Nordborg	CS22585
Ull2-5		Ullstorp-2	56.09	13.46	M. Nordborg	CS22586
Ull2-3			56.09	13.46	M. Nordborg	CS22587
RRS-7	U.S. Midwest (Indiana)	RRS	41.32	-86.26	J. Bergelson	CS22564
RRS-10			41.32	-86.26	J. Bergelson	CS22565
Knox-10		Knox	41.18	-86.38	J. Bergelson	CS22566
Knox-18			41.18	-86.38	J. Bergelson	CS22567
Rmx-A02	U.S. Midwest (Michigan)	Remax	42.07	-86.29	J. Bergelson	CS22568
Rmx-A180			42.07	-86.29	J. Bergelson	CS22569
Pna-17		Park/Napier	42.07	-86.27	J. Bergelson	CS22570
Pna-10			42.07	-86.27	J. Bergelson	CS22571
HR-5	England	HR	51.25	0.41	J. Bergelson	CS22596
HR-10			51.25	0.41	J. Bergelson	CS22597
NFA-8		NFA	51.25	0.41	J. Bergelson	CS22598
NFA-10			51.25	0.41	J. Bergelson	CS22599
Sq-1		SQ	51.25	0.41	J. Bergelson	CS22600
Sq-8			51.25	0.41	J. Bergelson	CS22601
CIBC-5		CIBC	51.25	0.41	J. Bergelson	CS22602
CIBC-17			51.25	0.41	J. Bergelson	CS22603
Uod-1	C. Europe (Austria)	Uod	48.07	14.53	M. Koch	CS22612
Uod-7			48.07	14.53	M. Koch	CS22613
Zdr-1	C. Europe (Czech Rep.)	Zdr	49.12	16.37	J. Relichová	CS22588
Zdr-6			49.12	16.37	J. Relichová	CS22589
Bor-1		Bor	49.12	16.37	J. Relichová	CS22590
Bor-4			49.12	16.37	J. Relichová	CS22591
Lp2-2		LP2	49.22	16.39	I. Cetl	CS22594
Lp2-6			49.22	16.39	I. Cetl	CS22595
Pu2-7	Croatia	Pu2	42.38	18.07	I. Cetl	CS22592
Pu2-23			42.38	18.07	I. Cetl	CS22593
Tamm-2	Finland	Tammisari	59.58	23.26	O. Savolainen	CS22604
Tamm-27			59.58	23.26	O. Savolainen	CS22605
Kz-1	Kazakhstan	KZ	49.5	73.1	I. Al-Shehbaz	CS22606
Kz-9			49.5	73.1	I. Al-Shehbaz	CS22607
Got-7	Germany	Göttingen	51.32	9.55	G Röbbelen	CS22608
Got-22			51.32	9.55	G Röbbelen	CS22609
Ren-1	France	Rennes	48.5	-1.41	G Röbbelen	CS22610
Ren-11			48.5	-1.41	G Röbbelen	CS22611
