# Supplementary material for: The Pattern of Polymorphism in Arabidopsis thaliana
Source: PLoS Biol. 2005 May 24;3(7):e196. doi: 10.1371/journal.pbio.0030196 (PMC1135296; doi:10.1371/journal.pbio.0030196)
Supplement: Supplementary file 2 [file pbio.0030196.st002.pdf]

Table S2. The accessions used in the project: individual accessions.

accession	region	population	latitude	longitude	origin	stock center
Cvi-0	Cape Verde Isl.	Cape Verde Isl.	16	-24	CS6675	CS22614
Lz-0	France	Puy-de-Dome	46	3.3	CS6788	CS22615
Ei-2	Germany	Eifel	50.3	6.3	CS6689	CS22616
Gu-0	Germany	Guckingen	50.3	8	CS6730	CS22617
Ler-1	Poland	Landsberg	52–53	15–16	CS6928	CS22618
Nd-1	Germany	Niederzenz	51 (NA)	10	CS6922	CS22619
C24	Portugal	Coimbra	40.12	-8.25	CS906	CS22620
CS22491	Russia	Konchezero	61.36	34.15	CS22491	CS22621
Wei-0	Switzerland	Weiningen	47.25	8.26	CS6182	CS22622
Ws-0	Ukraine	(Wassilewskija)/Djnepr	52.3	30	CS6891	CS22623
Yo-0	USA	Yosemite Nat. Park	37.45	-119.35	CS6901	CS22624
Col-0	USA (Germany?)	Columbia	38.3	-92.3	S. Somerville (CS6673)	CS22625
An-1	Belgium	Antwerpen	51.3	4.3	CS6603	CS22626
Van-0	Canada	UBC, Vancouver	49.3	-123	CS6884	CS22627
Br-0	Czech Republic	Brno (Brunn)	49	16.3	CS6626	CS22628
Est-1	Estland	Estland	58.3	25.3	CS6701	CS22629
Ag-0	France	Argentat	45	1.3	CS6601	CS22630
Gy-0	France	La Miniere	49	2	CS6732	CS22631
Ra-0	France	Randan, Puy-de-Dome	46	3.3	CS6844	CS22632
Bay-0	Germany	Bayreuth	49	11	CS6608	CS22633
Ga-0	Germany	Gabelstein	50.3	8	CS6714	CS22634
Mrk-0	Germany	Markt/Baden	49	9.3	CS6796	CS22635
Mz-0	Germany	Merzhausen/Ts.	50.3	8.3	CS6800	CS22636
Wt-5	Germany	Wietze	52.3	9.3	CS6896	CS22637
Kas-2	India	Kashmir	35	77	S. Somerville (CS6751)	CS22638
Ct-1	Italy	Catania	37.3	15	CS6674	CS22639
Mr-0	Italy	Monte/Tosso	44.3	9.3	CS6795	CS22640
Tsu-1	Japan	Tsushima	34.43	136.31	J. Borevitz (CS6926)	CS22641
Mt-0	Libya	Martubad/Cyrenaika	33	23	CS6799	CS22642
Nok-3	Netherlands	Noordwijk	52.3	4	CS6810	CS22643
Wa-1	Poland	Warzaw	52.3	21	S. Somerville (CS6885)	CS22644
Fei-0	Portugal	St. Maria d. Feiria	40 (NA)	-8	C. Alonso-Blanco	CS22645
Se-0	Spain	San Eleno	41.3	2.3	CS6852	CS22646
Ts-1	Spain	Tossa de Mar	41.3	3	CS6868	CS22647
Ts-5	Spain	Tossa de Mar	41.3	3	CS6871	CS22648
Pro-0	Spain	Proaza, Asturios	43.15	-6	C. Alonso-Blanco	CS22649
LL-0	Spain	Llagostera	41.59	2.49	C. Alonso-Blanco	CS22650
Kondara	Tajikistan	Pamiro-Alay	38.35	68.48	CS6175	CS22651
Shahdara	Tajikistan	Pamiro-Alay	38.35	68.48	CS6180	CS22652
Sorbo	Tajikistan	Pamiro-Alay	38.35	68.48	CS931	CS22653
Kin-0	USA	Kindalville, MI	44.46	-85.37	CS6755	CS22654
Ms-0	Russia	Moscow	56	38	CS6797	CS22655
Bur-0	Ireland	Burren	53.3	-8	CS6643	CS22656
Edi-0	Scotland	Edinburgh	56	-3	CS6688	CS22657
Oy-0	Norway	Oystese	60.23	6.13	CS6824	CS22658
Ws-2	Ukraine	(Wassilewskija)/Djnepr	52.3	30	CS2360	CS22659
